# Quercetin Arrests in G2 phase, Upregulates INXS LncRNA and Downregulates UCA1 LncRNA in MCF-7 Cells

**DOI:** 10.22088/IJMCM.BUMS.10.3.207

**Published:** 2022-01-10

**Authors:** Fatemeh Rezaie, Mohammad Javad Mokhtari, Mehdi Kalani

**Affiliations:** 1 *Department* * of Biology* *, Zarghan Branch, Islamic Azad University, Zarghan, Iran.*; 2 *Department of Immunology, Prof. Alborzi Clinical Microbiology Research Center, Shiraz University of Medical Sciences, Shiraz, Iran. *

**Keywords:** Breast cancer, quercetin, MCF-7 cells, *INXS*, *UCA1*

## Abstract

One of the most prevalent malignancies, which have severe effects on women's health, is breast cancer. Quercetin, a flavonoid found in vegetables, tea, and fruits, is known to have bioactive properties, such as anti-inflammatory, anti-oxidant, as well as anti-cancer. Long non-coding RNAs (lncRNAs) have been recognized to function as primary regulators of diverse cellular processes, including differentiation, development, and cell fate. *INXS* and *UCA1* are lncRNAs that are up regulated and down regulated respectively in cancer cells. This research aimed to assess the impact of quercetin on the expression of *INXS* and *UCA1* genes in MCF-7 cells. Various quercetin concentrations at different times were used to treat MCF-7 cells. The cell viability and IC_50_ values were determined using MTT assay. Then, MCF-7 cells were incubated with various quercetin concentrations for 24, 48, and 72 h. Cell cycle analyses were evaluated by flow cytometry. The levels of *INXS* and *UCA1* gene expression compared with the *GAPDH* gene at different concentrations of quercetin were quantified using real-time PCR method. Based on the results, quercetin exerted a dose- and time-dependent inhibitory impact on the viability of MCF-7 cells. Furthermore, quercetin induced cell cycle arrest at the G2 phase in MCF-7 cells. Also, quercetin induced *INXS* upregulation and *UCA1* downregulation in the MCF-7 cell line. These data suggest that quercetin might increase cell death by up regulating *INXS* and down regulating *UCA1* lncRNAs in MCF-7 cells.

In women, breast cancer is the most commonly diagnosed cancer and the leading cause of cancer death, followed by colorectal and lung cancer for incidence, and vice versa for mortality (1). Several methods exist for treating breast cancer, including chemotherapy and radiotherapy. However, such therapies have certain side effects on healthy cells (2).

It has been assessed that the human genome contains 23,000 long non-coding RNA (lncRNA) genes, which are more abundant than 20,000 protein-coding genes (3). LncRNAs are recognized as a new class of non-coding RNA that are longer than 200 nucleotides (4). They have an essential role in various malignancies. Recent researchers have shown that an array of long non-coding RNAs express abnormally in breast cancer. Although the molecular and the biological functions of lncRNAs remain enigmatic (5). Some of the lncRNAs are involved in the regulation of the apoptosis process. The upregulation of intronic BCL-XS-inducing (*INXS*) lncRNA results in a momentous activation in caspases 3, 7, and 9 in the intrinsic apoptosis pathway. It may decrease tumor size *in vivo*, hence have tumor suppressor features (6). Urothelial carcinoma associated 1 (*UCA1*) lncRNA triggers cell proliferation, migration, invasion, and cell apoptosis inhabitation (7).

Among the anti-cancer and cancer-preventing drugs, flavonoids may interfere with particular phases of the carcinogenic procedure, inhibit cell growth, and induce apoptosis in many kinds of cancer cells (8). One such natural flavonoid is quercetin (C_15_H_10_O_7_) that has multiple biological actions, such as anti-viral, anti-oxidant, anti-inflammatory, anti-cancer, apoptosis-inducing, and cell cycle modulatory and angiogenesis inhibitory impacts. It is abundantly found in diverse plant materials, as well as common foods and drinks, such as onions, apples, broccoli, berries, tomato, kale, cherry, and tea (9). When quercetin is consumed, it passes into the large intestine without any change (10). As in many cases, cancer treatment leads to poor outcomes (11), in the ongoing battle against cancer, the development of new therapeutic strategies remains an essential goal.

In this research, the effect of quercetin on MCF-7 cells viability was investigated. Further, the expression of *INXS* and *UCA1* genes and the cell cycle analysis was determined. Moreover, the association between the *INXS* and *UCA1* gene expressions with the cell cycle status was determined. Accordingly, it was attempted to assess the impacts of quercetin on the expression of *INXS* and *UCA1* lncRNAs genes for the first time.

## Materials and methods

**Cell culture**

The MCF-7 breast cancer cell line (12) was purchased from the National Cell Bank of Iran, Pasteur Institute of Iran, Tehran, Iran (NCBI No: C135). Recently, the MCF-7 cell line has been tested for mycoplasma contamination (13). MCF-7 cells were cultured in RPMI-1640 supplemented with 2 mM glutamine, 10% fetal bovine serum (FBS), and antibiotic (all from Gibco, Scotland), at 37 ^o^C in an incubator with 5% CO_2_ and 95% O_2_.

**Cell treatment and** **IC**_50_ **determination**

Using MTT assay, the cytotoxicity of quercetin was defined in the MCF-7 cell line. Quercetin (Sigma, USA) was dissolved in DMSO (Sigma, USA), In culture media, the DMSO under no circumstances exceeded 0.1% (v/v) (14). 1 × 10^4^ cells were seeded into 96-well plates in the full growth culture medium. Once every 12 h, the medium was replaced with 100 μL growth medium holding a series of different quercetin concentr- ations (0, 25, 50, 75, 150, and 300 μM) (15, 16) for 24, 48, and 72 h. From each well, the culture medium was detached after a specified period. Then, approximately 100 μL of the full medium with 20 μL MTT solutions were added to every well. The media were removed after 5 h incubation, and the crystals of formazan were solubilized for 10 min with 150 μL DMSO. Then, the formazan quantity was defined from the OD at 570 nm. The values of half-maximal inhibitory concentration (IC_50_) were defined through probit analysis applying Pharm PCS statistical package (Springer Verlag, New York, NY). To evaluate cell cycle and gene expression, the IC_50_ concentrations of quercetin were used to treat MCF-7 cells at different times.

**Flow cytometric analysis**

The viability of cells incubated with quercetin and stained with propidium iodide (PI) was measured using the flow cytometry method. Briefly, floating and trypsinized adherent cells were collected, and the harvested cells were aliquoted up to 1 × 10^6^ cells/100 μL into FACS tubes. The cells were washed twice by adding PBS up to 2 ml, centrifuging at 300 × g for 5 min, and decanting the buffer from the pelleted cells. Then, the cells were re-suspended in 100 µL staining buffer (PBS with 2% bovine serum albumin, 2 mM EDTA, and 2 mM sodium azide). Five microliters of PI staining solution (10 mg/mL) were added to the cells, mixed gently, and incubated on ice for 1 min in the dark. Then, the PI fluorescent cells were measured by flow cytometer instrument (FACSCalibure, BD, USA). The stained cells were gated based on the forward scatter (FSC) vs side scatter (SSC), and then the percentage of PI positive cells was measured on the FL3 channel. The frequency of PI^+^ cells, and the cell cycle phases was determined using FlowJo software version 10 https://www. flowjo.com/solutions/flowjo).

**RNA extraction, cDNA synthesis, and primer design**

RNA was isolated using Trizol solution. To remove DNA contamination, the isolated RNA was treated with DNase I enzyme. To evaluate the purity and concentration of the isolated RNA, a photo nanometer at 230, 260, and 280 nm was applied. RNA samples with the A260/A280 and A260/A230 ratios higher than 1.7 were chosen for cDNA synthesis. Up to 1 μg RNA was reversely transcribed into cDNA. cDNA synthesis was performed using the cDNA synthesis kit (Favorgen, Thailand) based on the manufacturer's instruction.

In the present study, the *GAPDH* gene was used as the reference, and *UCA1* and *INXS* genes were selected as target genes. Primer Express and Gene Runner software v.3.0 (Applied Biosystems, Foster City, CA) were used to design oligonuc-leotide primers and analyze them. Primers were confirmed through BLAST analysis to avoid non-specific PCR product formation (17).

The order of forward and reverse primers for the PCR amplification of *UCA1* and *INXS* transcripts was 5′-TTAGGCTGGCAACCATCAG ATC-3′ and 5′-TGTTGTCCTGGATGCTGGTCT-3′ (amplicon size, 127 bp) and 5′- TGATGTTGA AGGCCCGAGAC-3′ and 5′- AATCCCCAACTG CCACGTTC -3′ (amplicon size, 85 bp), respecti- vely.

The order of forward and reverse primers for *GAPDH* was 5′- CATGAGAAGTATGACAACAG CCT-3′ and 5′- AGTCCTTCCACGATACCAA AGT -3′, respectively (amplicon size, 113 bp).

**Quantitative real-time PCR**

The SYBR Green PCR Master Mix (Takara, Japan) was applied to carry out quantitative RT-PCR on the Rotor-Gene 6000 (Corbett Research, Australia) with the thermal-cycling settings of 10 min at 95 ^o^C followed by 40 cycles of 15 s at 95^ o^C and 1 min at 60^ o^C. Every complete amplification phase was accompanied by a melting phase. To determine non-specific amplification, melting curves were used in this study. Non-template controls (NTC) were included in each run. By plotting Ct values facing log cDNA concentrations of five serial two-fold dilution, a dynamic range of the target and reference genes were assessed to compute the PCR efficiency. The response efficacy was computed via the equation below: E = [10^(-1/slope^)–1] (18). The *GAPDH* gene was selected to normalize target genes’ expression. 2^-ΔΔCT^ was used to calculate the mRNA comparative expression level of each target gene (19). 

**Statistical analysis**

The statistical data, including mean ± SD and correlation coefficients (R^2^), graph preparation, were performed using Microsoft office excel 2010 software. In this work, the statistical significance of differences was calculated via a Student’s *t-*test, together with one-way variance analysis. The P value <0.05 was regarded as statistically significant.

## Results

**Quercetin cytotoxicity and IC**_50_ **determination**

**Fig.1:**
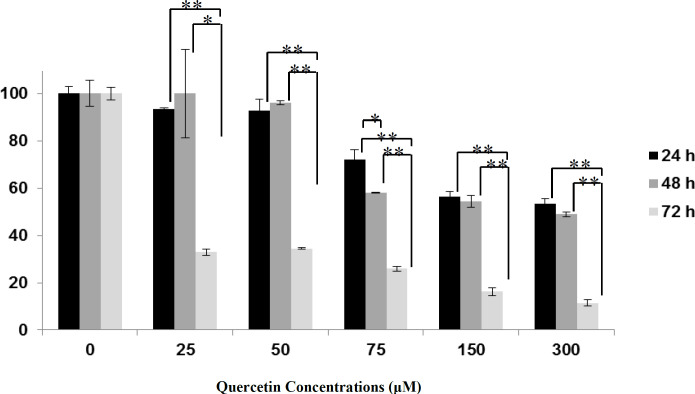
**Concentration- and time-dependent effects of quercetin on MCF-7 cells** **viability. **Results are expressed as the percentage of viability compared with the control, and are presented as mean±SD. Data were analyzed by one-way ANOVA. Significance was set at *P < 0.05, **P < 0.001, *** P < 0.0001

In MCF-7 cells, the cytotoxicity of quercetin was evaluated by MTT assay. Cells were incubated with various quercetin doses for 24, 48, and 72 h. Cytotoxic effects of the quercetin concentrations are illustrated in figure 1. Data show that quercetin decreases the viability of MCF-7 cells dose-dependently. Compared to the controls, the lower dose of quercetin (25 μM) decreased the total cell number about 6.59% ± 0.60 (P> 0.05), 0.11% ± 18.70 (P> 0.05) and 67.87% ± 1.39 (P <0.001) after 24, 48 and 72 h, respectively, while its higher dose (300 μM) decreased the total cell number about 46.76% ± 2.44 (P <0.01), 51.00% ± 1.00 (P <0.01) and 88.64% ± 1.35 (P <0.001) after 24, 48 and 72 h, respectively. For 25, 50, 75, 150, and 300 μM quercetin, significant differences in cell viability were observed at 24 h vs 72 h and 48 h vs 72 h. For 75 μM quercetin significant differences were observed at 24 h vs 48 h after treatment (P <0.05). The IC_50_ of quercetin after 24, 48, and 72 h was 265 µΜ, 150 µΜ, and 10 µΜ, respectively.

**Induction of cell cycle arrest by quercetin**

**Fig.2:**
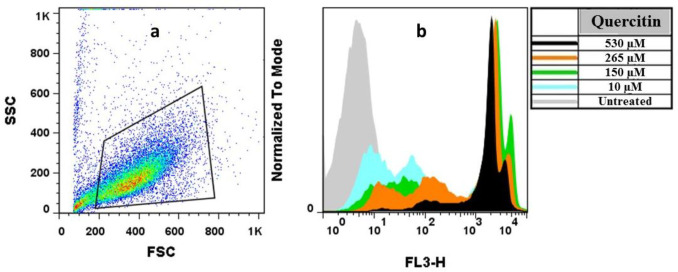
**Flow cytometry analysis for MCF-7 cells treated by different concentrations of quercetin. **a) MCF7 cells were gated based on the forward scatter (FSC) vs. side scatter (SSC); b) the effects of different concentrations of quercetin on cell viability was determined among the gated populations stained with propidium iodide

**Fig.3:**
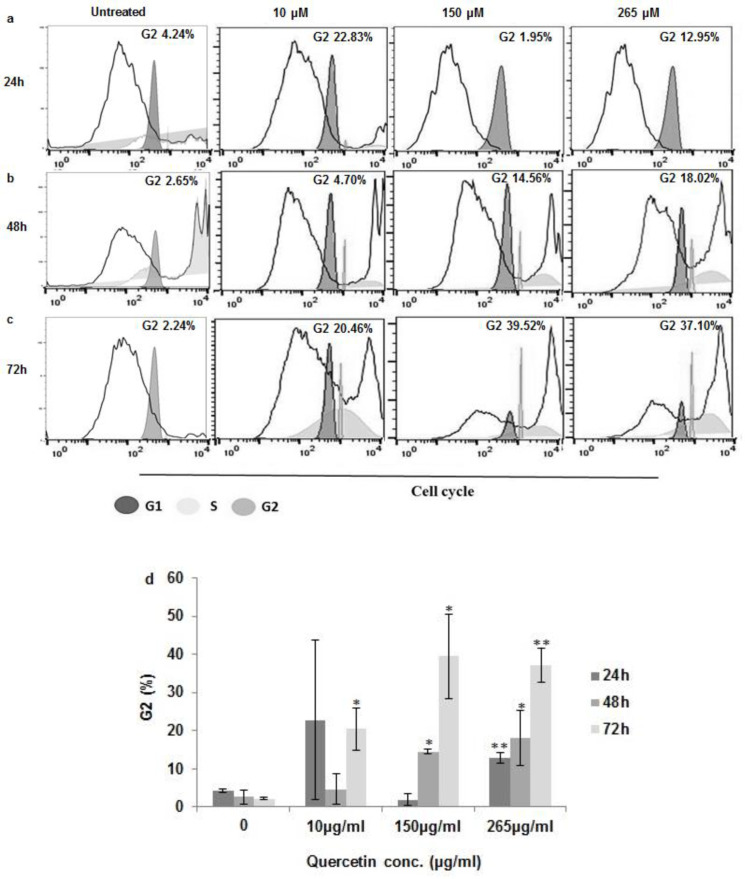
**Cell cycle arrest by quercetin.** MCF-7 cells incubated with various concentrations of quercetin (a) at 24h, (b) 48h and, (c) 72h cells were stained with propidium iodide to determine cell cycle distribution by FACS flow cytometry. No treated cells were used as control. (d) Statistical significances of difference throughout this research were computed using one-way variance analysis. Values with the asterisks are significantly different from the controls *P<0.05, **P<0.001

Quercetin has proven multiple biological activities on cell viability and cell cycle. For this purpose quercetin-treated MCF7 was stained with PI and was analyzed via flow cytometry (Figure 2) to examine whether the quercetin-induced toxicity is due to its impact on cell viability and proliferation. The findings showed that quercetin for 24 h did not induce significant changes in MCF-7 cells at 10 and 150 μM, while at 265 μM, a significant increase in the G2/M phase cells was reached (Figure 3a, 3d). Furthermore, quercetin induced significant changes in MCF-7 cells at 150 and 265 μM for 48 h and also significant changes in MCF-7 cells at 10, 150, and 265 μM for 72 h (Figure 3bd).

**Real-time PCR validation**

**Fig.4:**
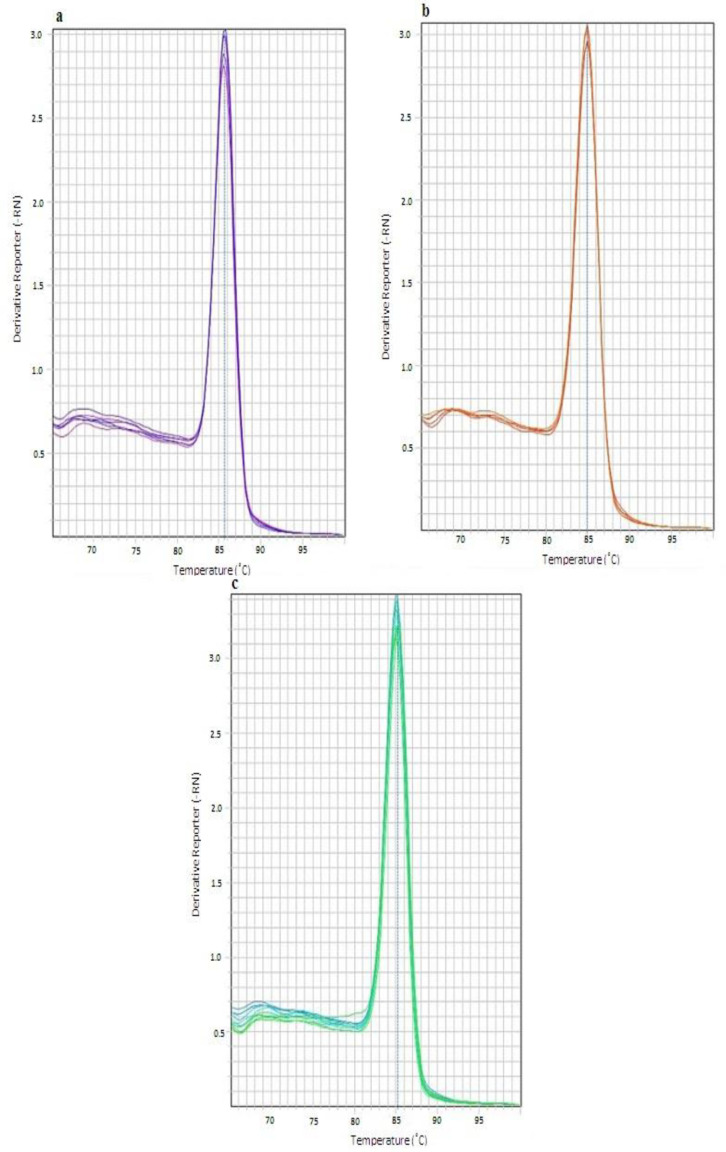
**The melting analysis of the Real time PCR assays. **Melting peaks at (a) 85.88°C for *UCA1* gene, (b) 84.93°C for *INXS* gene and (c) 85.23 for *GAPDH* gene indicate the formation of the tree specific products with different Tm temperatures

Melting curve analysis was used to confirm the formation of a single amplicon for every gene. The melting peaks were achieved at 85.88°C for the* UCA1* gene, 84.93 °C for the *INXS *gene, and 85.23 for the *GAPDH* gene (Figure 4)

**Fig.5:**
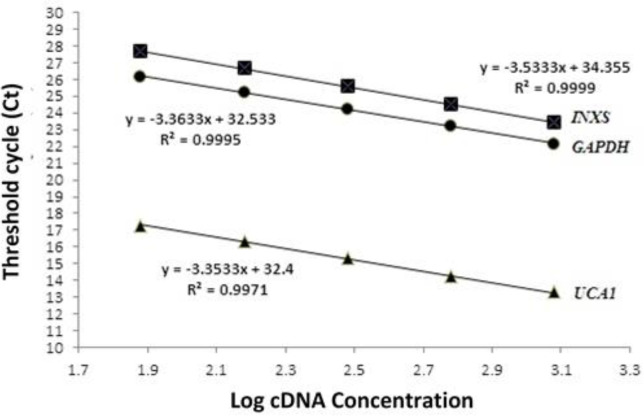
**Standard curve for INXS, UCA1 and GAPDH. **Standard curve of INXS, slope=−3.53, y-intercept=34.35, R2=0.999. Standard curve of UCA1, slope=−3.35, y-intercept= 32.4, R2=0.997. Standard curve of GAPDH, slope=−3.36, y-intercept= 32.53, R2=0.999

According to the initial data, the expression differences between control and target genes in the treated samples were not more than tenfold. Thus, in the standard curves, two-fold serial dilution was used to confirm the test accuracy. This allowed us to increase the dynamic range resolution. The slope of the standard curves was -3.35, -3.53, and -3.36 for *UCA1*, *INXS*, and *GAPDH*, respectively. The efficiency of PCR was computed as 98.60% for *UCA1*, 91.86% for *INXS*, and 98.15% for *GAPDH *(Figure 5).

**Fig.6:**
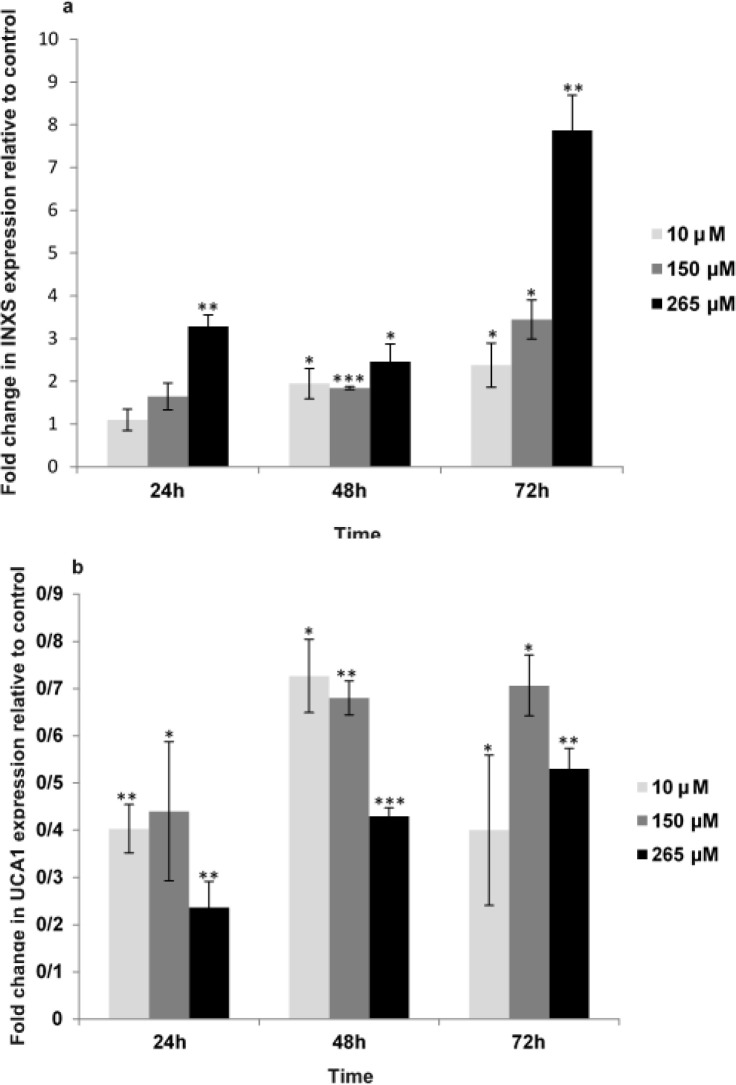
(a) Quercetin effect on LncRNA INXS expression. (b) Quercetin effect on LncRNA UCA1 expression. Bars represent fold differences of the mean of normalized expression values ± SD. Values with asterisk are significantly different from the controls *P<0.05, **P<0.001, *** P<0.001

**Relative quantification of** ***INXS*** **and** ***UCA1*** **expression**

The relative gene expression among two samples (treated and untreated) was defined using the difference in their Ct values. The mCt value for *GAPDH* was 24.35 ± 0.61 in various quercetin concentrations. The *INXS*/*GAPDH* and *UCA1*/ *GAPDH* gene expression ratios in MCF-7 cells treated with different quercetin concentrations (10, 150, 265 µΜ) was obtained through the 2^-ΔΔCT ^formula. The most *INXS*/*GAPDH* ratio was computed as 7.87 for 72 h (Figure 6a), and the less *UCA1*/*GAPDH* ratio was calculated as 0.23 for 24 h (Figure 6b).

## Discussion

Very little is known about the association between lncRNAs and dietary factors. In this research, *INXS* and *UCA1* lncRNAs expression were investigated in MCF-7 cells treated with quercetin. Our data revealed that lncRNAs levels may change by treatment with quercetin. In a time and dose-dependent manner, quercetin treatment decreased the viability of MCF-7 cells. The induction of G2 phase arrest in the MCF-7 cells was confirmed by flow cytometric analysis. These changes were in association with the upregulation of *INXS* and the downregulation of *UCA1* expressions. To the author's knowledge, the present study is the first to explore the quercetin effects on the expression of *INXS* and *UCA1* lncRNAs. Diet has a crucial role in maintaining a healthy life. Our daily diet may contain some products like flavonoids, which can prevent cancer progression. In recent years, chemists and dietitians found that quercetin is a dietary component and a distinctive bioactive flavonoid, that has various health‐promoting impacts (20). Epidemiologic research has proposed that high flavonoid consumption might correlate with a reduced risk of different types of tumors (21). Quercetin also shows direct pro-apoptotic impacts on cancer cells, which may prevent the progression of many human cancers (20). The anti-cancer effects of quercetin have been documented in many previous investigations involving cell lines and animal models. On the other hand, quercetin has minimal damages or side effects on healthy cells despite its high-toxic impact against tumor cells (21).

Some studies have shown that the incubation of breast cancer cell lines with quercetin leads to G1 phase arrest together with apoptosis (20, 22). Furthermore, quercetin has demonstrated an inhibitory effect on MCF‐7 and MDA‐MB‐231 cells through different mechanisms, including the upregulation of miR‐146a expression and activation of caspase‐3 (23). Quercetin also can cause cell cycle arrest and induce apoptosis by modulating pro-apototic, PI3K/Akt, cyclins and mitogen-activated protein kinase (MAPK) molecular pathways (24). Seo *et al*. found that, quercetin inhibits the clonogenic survival and proliferation of breast tumor cells. These growth inhibitions were accompanied with an increase in sub-G0/G1 apoptotic populations. Also, quercetin could induce the upregulation of cleaved caspase‐3 and ‐8, resulting in the poly ADP ribose polymerase (PARP) cleavage. However, it did not influence the levels of BAX and BCL‐2, and did not induce apoptosis via the intrinsic apoptosis pathway (25).

In the present study, quercetin decreased cell viability through G2 phase arrest. The investigation by Zhao et al. found that in colon cancer cells, quercetin triggered G2 phase cell cycle arrest. It also induced autophagic cell death via extracellular-signal-regulated kinase stimulation (26, 27). In MDA-MB-231 cells, quercetin induces apoptosis and cell cycle arrest (28). Incubation of 143B cell line with quercetin noticeably led to proliferation inhibition, apoptosis promotion, and G2/M phase cell cycle arrest (29). Quercetin might raise the proportion of cells in the S and G2 phases, and decline the ratio of the G0/G1 phase cells. Down regulation of nuclear factor‐κB and *BCL‐2* genes, upregulation of the *BAX* gene, activation of caspase‐3, and promotion of leukemic cell apoptosis, are other outcomes of quercetin function. On the other hand, it was shown that quercetin might reduce leukemia cell proliferation and promote apoptosis (30). Quercetin was also shown to inhibit cell viability, induce DNA damage , promote G2-M cell cycle arrest, and activate both apoptotic pathways in cervical cancer cells (31). 

In the present study, quercetin treatment upregulated the *INXS* lncRNA, and downregulated the *UCA1* lncRNA in the MCF-7 cells. This is the first research to our knowledge, which investigates the effect of quercetin treatment on *INXS* and *UCA1* lncRNAs gene expressions in this cell line. Many reports specify that lncRNAs may regulate apoptosis through different patterns and levels (32). There are a few instances of dietary effects on lncRNAs expression. In a recent study, we have evaluated the expression of *MALAT1* and *MIAT* lncRNA genes in HUVEC cells treated with quercetin. Quercetin treatment reduced the viability of HUVEC cells in a dose- and time-dependent manner. We also observed the downregulation of *MALAT1* and *MIAT* lncRNAs in HUVEC cells treated with quercetin. Thus, this study suggested that quercetin can inhibit angiogenesis via *MALAT1* and *MIAT* downregulation (33). *INXS* is an lncRNA that can shift the *BCL-X* alternative splicing from the anti-apoptotic *BCL-XL* to the pro-apoptotic *BCL-XS*. *INXS* up regulation leading to the accumulation of BCL-XS, activation of caspases, and induction of apoptosis (34). The *UCA1* lncRNA overexpresses wingless-type MMTV integration site family member 6, enhancing the BAX inhibition via protein kinase B and conferring resistance to cisplatin-induced apoptosis (35, 36). *UCA1* silencing in LLC9 and LLC2 cell lines increases tamoxifen drug sensitivity by inducing apoptosis and arresting the G2/M phase cell cycle. Notably, the induced upregulation of *UCA1* in T47D and MCF7 breast cancer cells decreases the tamoxifen drug sensitivity (37). Some reports explored how various dietary patterns change the expression of *H19* lncRNA that regulates cell proliferation (38, 39). In an additional study, in colorectal cancer, resveratrol was shown to regulate the Wnt/β-catenin signaling pathway by downregulating *MALAT1 *(40).

Here, we showed that quercetin upregulates the *INXS* expression and downregulates the *UCA1* expression in the MCF-7 cell line. Phytochemicals, such as quercetin with their regulatory effects on lncRNAs, may be helpful as a natural component for different cancer therapies. Significantly, they are essential natural compounds with no signs of side effects or toxicity.

Our study has some limitations as only one human breast cancer cell line was studied. Therefore, the obtained results cannot be generalized to other cell lines, and the clinical outcomes should be interpreted with caution. Also, in the current investigation, we evaluated the expression of *INXS* and *UCA1* lncRNAs without performing a full transcriptome analysis.

## Conflict of Interest

The authors state no conflict of interest.
